# A Neurometabolic Pattern of Elevated Myo-Inositol in Children Who Are HIV-Exposed and Uninfected: A South African Birth Cohort Study

**DOI:** 10.3389/fimmu.2022.800273

**Published:** 2022-03-28

**Authors:** Cesc Bertran-Cobo, Catherine J. Wedderburn, Frances C. Robertson, Sivenesi Subramoney, Katherine L. Narr, Shantanu H. Joshi, Annerine Roos, Andrea M. Rehman, Nadia Hoffman, Heather J. Zar, Dan J. Stein, Kirsten A. Donald

**Affiliations:** ^1^ Department of Paediatrics and Child Health, Red Cross War Memorial Children’s Hospital, University of Cape Town, Cape Town, South Africa; ^2^ Research Master Brain and Cognitive Sciences, Faculty of Science, University of Amsterdam, Amsterdam, Netherlands; ^3^ Department of Clinical Research, London School of Hygiene & Tropical Medicine, London, United Kingdom; ^4^ Neuroscience Institute, University of Cape Town, Cape Town, South Africa; ^5^ Department of Human Biology, University of Cape Town, Cape Town, South Africa; ^6^ Cape Universities Body Imaging Centre (CUBIC), Cape Town, South Africa; ^7^ Departments of Neurology, Psychiatry and Biobehavioral Sciences, University of California, Los Angeles, Los Angeles, CA, United States; ^8^ SAMRC Unit on Risk and Resilience in Mental Disorders, Stellenbosch University, Cape Town, South Africa; ^9^ MRC International Statistics & Epidemiology Group, London School of Hygiene & Tropical Medicine, London, United Kingdom; ^10^ Department of Psychiatry and Mental Health, University of Cape Town, Cape Town, South Africa; ^11^ SAMRC Unit on Child & Adolescent Health, University of Cape Town, Cape Town, South Africa; ^12^ SAMRC Unit on Risk and Resilience in Mental Disorders, University of Cape Town, Cape Town, South Africa

**Keywords:** HIV exposure, magnetic resonance spectroscopy, neuroinflammation, brain development, myo-inositol

## Abstract

**Introduction:**

Exposure to maternal HIV in pregnancy may be a risk factor for impaired child neurodevelopment during the first years of life. Altered neurometabolites have been associated with HIV exposure in older children and may help explain the mechanisms underlying this risk. For the first time, we explored neurometabolic profiles of children who are HIV-exposed and uninfected (CHEU) compared to children who are HIV-unexposed (CHU) at 2-3 years of age.

**Methods:**

The South African Drakenstein Child Health Study enrolled women during pregnancy and is following mother-child pairs through childhood. MRI scans were acquired on a sub-group of children at 2-3 years. We used single voxel magnetic resonance spectroscopy to measure brain metabolite ratios to total creatine in the parietal grey matter, and left and right parietal white matter of 83 children (36 CHEU; 47 CHU). Using factor analysis, we explored brain metabolite patterns in predefined parietal voxels in these groups using logistic regression models. Differences in relative concentrations of individual metabolites (n-acetyl-aspartate, myo-inositol, total choline, and glutamate) to total creatine between CHEU and CHU groups were also examined.

**Results:**

Factor analysis revealed four different metabolite patterns, each one characterized by covarying ratios of a single metabolite in parietal grey and white matter. The cross-regional pattern dominated by myo-inositol, a marker for glial reactivity and inflammation, was associated with HIV exposure status (OR 1.63; 95% CI 1.11–2.50) which held after adjusting for child age, sex, and maternal alcohol use during pregnancy (OR 1.59; 95% CI 1.07 –2.47). Additionally, higher relative concentrations of myo-inositol to total creatine were found in left and right parietal white matter of CHEU compared to CHU (p=0.025 and p=0.001 respectively).

**Discussion:**

Increased ratios of myo-inositol to total creatine in parietal brain regions at age 2-3 years in CHEU are suggestive of early and ongoing neuroinflammatory processes. Altered relative concentrations of neurometabolites were found predominantly in the white matter, which is sensitive to neuroinflammation, and may contribute to developmental risk in this population. Future work on the trajectory of myo-inositol over time in CHEU, alongside markers of neurocognitive development, and the potential for specific neurodevelopmental interventions will be useful.

## Introduction

Human immunodeficiency virus (HIV) infection remains a major public health concern worldwide, with 37.7 million people reported to be living with HIV globally (1). Of these, an estimated 25.3 million people live in sub-Saharan Africa. The widespread roll-out of antiretroviral therapy (ART) and expansion of ART programmes for prevention of mother-to-child transmission (PMTCT) have led to dramatic declines in vertical transmission rates to less than 5% during recent years (2). Globally, the estimated number of new infections in children aged 0 to 14 years has decreased by more than 60% since the year 2000 (3). However, progress in the eradication of paediatric HIV infection has revealed a concern that children who are HIV-exposed and uninfected (CHEU) remain a vulnerable population (2, 4). Approximately 15.4 million children worldwide are CHEU, 13.8 million of whom live in sub-Saharan Africa (1), with the highest number of CHEU residing in South Africa (3). Due to expanding accessibility of both ART and PMTCT programmes this population is increasing in number, however, the implications of HIV and ART exposure as risk factors for long-term child health and development are less well defined (4, 5).

Meta-analyses have found that CHEU are at a greater risk of all-cause mortality and worse developmental outcomes within the early years of life, compared to children who are HIV-unexposed (CHU) (6, 7). In sub-Saharan Africa, recent studies have described HIV exposure to be associated with neurodevelopmental delay (8–11) in children younger than 3 years of age. However, there is inconsistency across studies and settings, and others have reported CHEU having similar outcomes to CHU (12, 13).

There are a number of hypothesised mechanisms by which HIV exposure may impact paediatric brain development. As argued in the two-hit model of early brain damage, inflammatory intrauterine conditions may increase vulnerability of the developing brain to postnatal adverse events (14, 15). Since chronic inflammation can persist in HIV infection despite ART, women living with HIV may have immune dysregulation during pregnancy (16, 17). This may prime the developing brain to trigger exaggerated inflammatory responses against future insults, compromising typical neurobiological development (18–20). Immunological studies suggest the immune system of CHEU is altered compared to that of CHU (17, 21), some revealing proinflammatory immune profiles from birth to 2 years of age (22, 23). Neurobiological development in CHEU may therefore be affected by maternal immune dysregulation during pregnancy, however, studies of early neurometabolic development are lacking.

Exposure to ART has also been associated with potential neurotoxicity (24). Although maternal ART and child prophylaxis are important to prevent HIV transmission, potential metabolic and neurological consequences have been reported (25). Furthermore, environmental stressors are known to influence long term neurodevelopmental outcomes during the period from conception to 2 years of age, and psychosocial risk factors such as maternal antenatal depression and alcohol use in pregnancy may play a key role in child development (26, 27). Overall, there remains a gap in understanding the neurobiological consequences of HIV exposure in the context of high-risk environments.

Neuroimaging studies provide a key opportunity to examine HIV exposure-related neuropathophysiology (28), with reports describing white matter and grey matter differences between newborns who are HEU compared to HU (29, 30) and white matter abnormalities in older children who are HEU (31). Amongst the existing techniques, magnetic resonance spectroscopy (MRS) is a powerful approach, since it provides *in vivo* measurements of neurometabolites in specified brain regions. MRS profiles of the neurotypical brain during childhood are well characterized (32, 33), and this technique has previously been used to describe metabolite alterations in children older than 2 years with perinatal infection or exposure to HIV (34–36). Only one cohort study to date has examined neurometabolic characteristics of CHEU, reporting metabolite alterations in the basal ganglia at age 9 years, and in the frontal grey matter (GM) and peritrigonal white matter (WM) at age 11 years, compared to CHU (35, 36). MRS data are suitable for dimensionality reduction methods like factor analysis, which groups similar variables into a smaller number of dimensions. Through the combination of metabolite measurements across different brain regions, this method identifies metabolic patterns that underlie latent neurobiological processes. Factor analysis has previously been used in MRS studies to identify metabolic patterns within the context of HIV-related illness (36–38).

The aim of our study was to explore differences in brain metabolites in a well-characterized cohort of CHEU and CHU from similar sociodemographic conditions at 2-3 years of age, using MRS and factor analysis. We hypothesised that CHEU would have altered neurometabolic profiles compared to CHU in GM and WM, related to factors associated with inflammation.

## Methods

### Participants

The Drakenstein Child Health Study (DCHS) is a population-based birth cohort study in a peri-urban area of the Western Cape, South Africa, focused on investigating the early-life determinants of child health, development and illness (39–41). The local population is a low socioeconomic community with a high prevalence of several health risk factors including HIV infection.

The DCHS enrolled pregnant women between 2012 and 2015 during their second trimester of gestation and currently follows the mother-child pairs into middle childhood. Inclusion criteria for enrolment were a minimum age of 18 years, gestational period of 20–28 weeks, planned attendance at one of the two clinics and intention to remain in the area. All mothers gave written informed consent.

A subset of children enrolled in the DCHS participated in a longitudinal neuroimaging sub-study. As part of the neuroimaging sub-study, children who had undergone neonatal imaging (41) were invited to be scanned at 2-3 years. In addition, children not imaged at birth were also included selecting for risk factors (maternal HIV and alcohol use during pregnancy) to ensure a representative sample of a high-risk population, along with a randomly selected comparison group. These children were currently active in the study and living in the area. Exclusion criteria applied to children for this sub-study were: medical comorbidities such as congenital abnormality, genetic syndrome, or neurological disorder; low Apgar score (<7 at 5 minutes); neonatal intensive care admission; history of maternal use of illicit drugs during pregnancy; child HIV infection; and MRI contra-indications including cochlear implants (42).

### Sociodemographic Data Collection

The HIV status of enrolled mothers was confirmed *via* routine testing during pregnancy and re-checked every 12 weeks, in accordance with the Western Cape PMTCT guidelines (43). Children who were HIV-exposed were tested at age 6 weeks, 9 months, and 18 months using PCR, rapid antibody, or ELISA tests as per guidance. CHEU were confirmed to be negative for HIV at the age of 18 months, or once the mother had stopped breastfeeding if this lasted more than 18 months. CHU were defined as children born to mothers without HIV infection. Mothers living with HIV received ART according to PMTCT guidelines at the time. CHEU were prescribed post-exposure prophylaxis from birth (44). Maternal CD4 cell count and viral load data during pregnancy were abstracted from clinical records and the online National Health Laboratory Service system, collected as part of clinical care protocols. The lowest maternal CD4 cell count within 1 year before child’s birth and 3 months after birth was used to maximise numbers.

Sociodemographic and maternal psychosocial data were collected between weeks 28 and 32 of gestation, through interviews and questionnaires adapted from the South African Stress and Health study (39, 40). Infant birthweight and markers of poor nutrition were also collected, in accordance with the World Health Organization (WHO) Z-score guidelines (45). Stunting was defined as low child height-for-age, underweight as low child weight-for-age, and wasting as low child weight-for-length, all calculated as Z-scores lower than -2 of the WHO Child Growth Standards median. Maternal alcohol use during pregnancy was assessed using the Alcohol, Smoking, and Substance Involvement Screening Test (ASSIST), and data on moderate-severe alcohol use in pregnancy was retrospectively collected, forming a dichotomous measure (41). Maternal smoking during pregnancy was determined through self-reporting. Maternal depression was assessed with the Edinburgh Postnatal Depression Scale.

### Magnetic Resonance Spectroscopy Protocol

Participants in the neuroimaging sub-study underwent a multimodal magnetic resonance imaging (MRI) protocol without sedation, performed between January 2016 and September 2018 at Groote Schuur Hospital, University of Cape Town, on a 3 Tesla Siemens Skyra 70cm diameter bore whole body MRI scanner (Erlangen, Germany) using a 32-channel head coil (42). Once informed consent was acquired from the mother and the child had fallen into deep sleep, children were carried into the scanner, positioned carefully with pillows, blankets, and ear protection. MRS data acquisition was performed during natural sleep, and a trained study staff member remained in the scanner room during the entire session in case the child woke (42).

**Figure 1:**
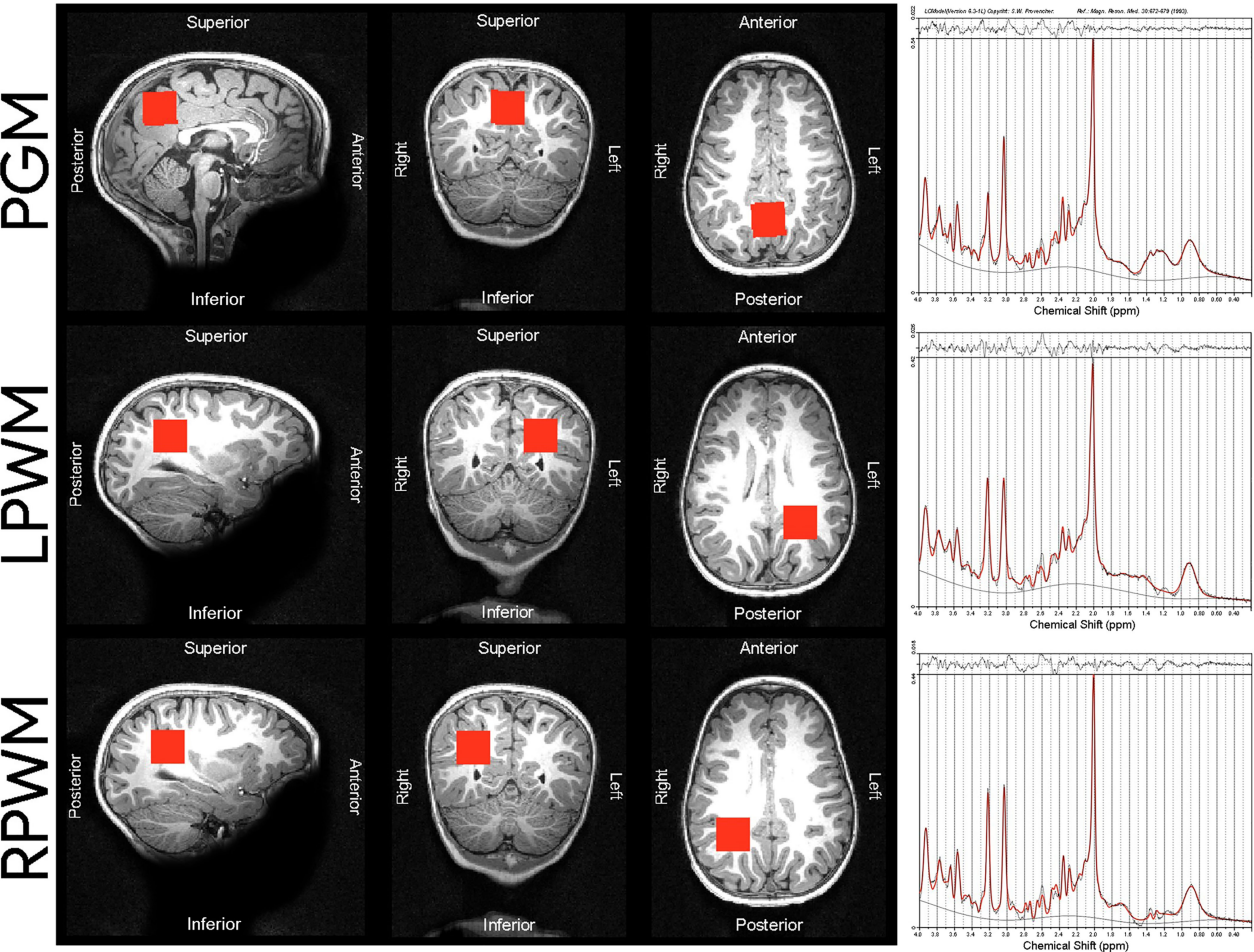
Voxel placements and sample spectra of median SNR and LCModel output from the 3T scanner used in our cohort of children. The following median SNR were obtained for each voxel placement: PGM median SNR = 34, LPWM median SNR = 35, RPWM median SNR = 33. PGM, parietal grey matter; LPWM, left parietal white matter; RPWM, right parietal white matter; SNR, signal-to-noise ratio; ppm, parts per million.

The MRS protocol was performed by well-trained radiographers who were blinded to the children’s HIV exposure status. It consisted of a high-resolution T1-weighted multi-echo magnetisation prepared rapid gradient echo acquisition (MEMPRAGE (46); sagittal orientation, repetition time (TR) 2530 ms, echo times (TE) = 1.69/3.54/5.39/7.24 ms, flip angle 7.0°, voxel size 1.0 x 1.0 x 1.0 mm^3^, inversion time (TI) 1100 ms, field of view (FOV) 224 x 224 x 176 mm, 176 slices, scan time 5 min 21 s) and single voxel Point RESolved Spectroscopy (PRESS; TR 2000 ms, TE 30 ms, 128 averages, voxel size 25 x 25 x 25 mm^3^, vector size 1024, spectral bandwidth 1200 Hz, scan time 6 min) with Chemical Shift Selective (CHESS) water suppression. A water reference was acquired without using CHESS. Shimming was automatically performed over the voxel volume (with use of the scanner’s advanced adjustments) and manually adjusted if necessary, to reduce the spectral linewidths reported by the scanner. Voxel 1 was targeted at the midline parietal GM, voxels 2 and 3 were targeted at left and right parietal WM respectively (**Figure 1**).

### Magnetic Resonance Spectroscopy Data Processing

MRS voxels were registered to the T1-weighted structural image with use of MATLAB software (MATLAB. Natick, Massachusetts: The MathWorks Inc.; 2017). Segmentation of the structural image into GM, WM, and cerebrospinal fluid (CSF) was performed using Statistical Parametric Mapping (SPM12) software (www.fil.ion.ucl.ac.uk/spm) to determine tissue composition for each voxel.

LCModel software (version 6.3-1) (47) was run to fit the raw spectral data for quantification, using the appropriate water reference for eddy current correction. Relative concentrations (ratios) to the reference signal, creatine and phosphocreatine (Cr+PCr), were determined for n-acetyl-aspartate (NAA/Cr+PCr), myo-inositol (Ins/Cr+PCr), total choline (glycerophosphocholine and phosphocholine, GPC+PCh/Cr+PCr), and glutamate (Glu/Cr+PCr). Quality of spectra was inspected visually and assessed in terms of full width at half maximum (FWHM) and signal-to-noise ratio (SNR), and Cramér-Rao lower bounds (CRLB) given by LCModel. Spectra with FWHM values greater than 0.08, and SNR values lower than 10 were considered of low quality and therefore excluded.

The four metabolites considered in our study have been characterized in terms of clinical significance in prior studies, from birth through childhood (32, 33). N-acetyl-aspartate is most commonly considered to be a marker for neuronal health or density in the developing brain (32, 33). While we note that the role of n-acetyl-aspartate in mature brain remains to be fully established and recognise that n-acetyl-aspartate may also play additional roles, such as contributing to myelin synthesis in the mature brain (48), the evidence for this is currently limited. Myo-inositol is considered a marker for glial reactivity, gliosis and neuroinflammation. Total choline is associated with myelination, membrane synthesis and membrane maturation in the WM. Glutamate, the main excitatory neurotransmitter in the brain, is considered a marker for neuronal function involved in many neurobiological and behavioural processes during brain development (32, 33).

### Statistical Analysis

Sociodemographic characteristics of the mother-child pairs were reported as mean (± SD) for continuous data, or absolute frequencies (%) for categorical data. Continuous data was assessed for normality using Shapiro-Wilk tests. Comparisons between CHEU and CHU were made using *t*-tests or Wilcoxon tests for normally and non-normally distributed continuous data, respectively, and X^2^ tests for categorical data.

Factorability of MRS data was assessed using Bartlett sphericity and Kaiser-Meyer-Olkin (KMO) tests. Factor analysis was carried out with use of a maximum likelihood approach and varimax rotation, and Root Mean Square Errors of Approximation (RMSEA) of less than 0.05 were considered to indicate statistical goodness of fit of the model. As proposed by Yiannoutsos and colleagues (38), factor scores were constructed for MRS data using a weighted linear combination of all 12 variables (the ratios of 4 metabolites to total creatine in each of the 3 voxels), multiplying each metabolite concentration by its associated factor loading and summing all products to form each of four factor scores (38).

To determine whether the brain metabolic patterns could predict HIV exposure, the factor scores obtained from brain metabolite ratios were included as independent variables in logistic regression models, to estimate odds ratios (OR) and 95% confidence intervals (CI). Both unadjusted and multivariable models were created. Potential confounders were chosen *a priori* due to their reported influence in neurometabolic or neurobehavioral outcomes in children. These included child age (32, 33), child sex (27, 49), and maternal alcohol use during pregnancy (50, 51).

Sensitivity analyses were performed to examine the effect of sociodemographic characteristics that showed significant differences (p<0.05) between CHEU and CHU, by additionally adjusting for these variables: maternal age of delivery, and maternal depression during pregnancy. Despite having similar values between groups, infant birthweight was also included in the sensitivity analysis, since its role as confounder or mediator in the causal pathway of maternal HIV infection and child developmental outcomes may vary across settings (52).

Region-specific analyses were run for each metabolite ratio, to explore differences between CHEU and CHU. Comparisons between groups were made using unadjusted and adjusted linear regression analyses with robust standard errors. Child age, child sex, and maternal alcohol use during pregnancy were included as covariates. To account for the presence of GM in voxels targeted at parietal WM, GM percentage was included as a confounder in sensitivity analyses.

Lastly, we planned to examine the association of each child metabolite pattern identified from factor analysis, with maternal immune status during pregnancy and time of maternal ART initiation, using multinomial logistic regression to estimate relative risk ratios. For maternal immune status during pregnancy, a categorical variable was created with the following levels: lowest maternal CD4 cell count during pregnancy ≤500 cells/mm^3^ versus >500 cells/mm^3^ in CHEU. Similarly, for maternal ART initiation, a categorical variable was created examining maternal ART initiation before pregnancy versus during pregnancy. CHU was used as the reference in both models. A Cramér’s V test was run to check for multicollinearity between the categorical variables.

Statistical analyses were performed in R with RStudio software (version 1.2.5033) (53). P values of less than 0.05 (two-tailed) were considered statistically significant.

## Results

### Cohort and Demographic Characteristics

**Figure 2:**
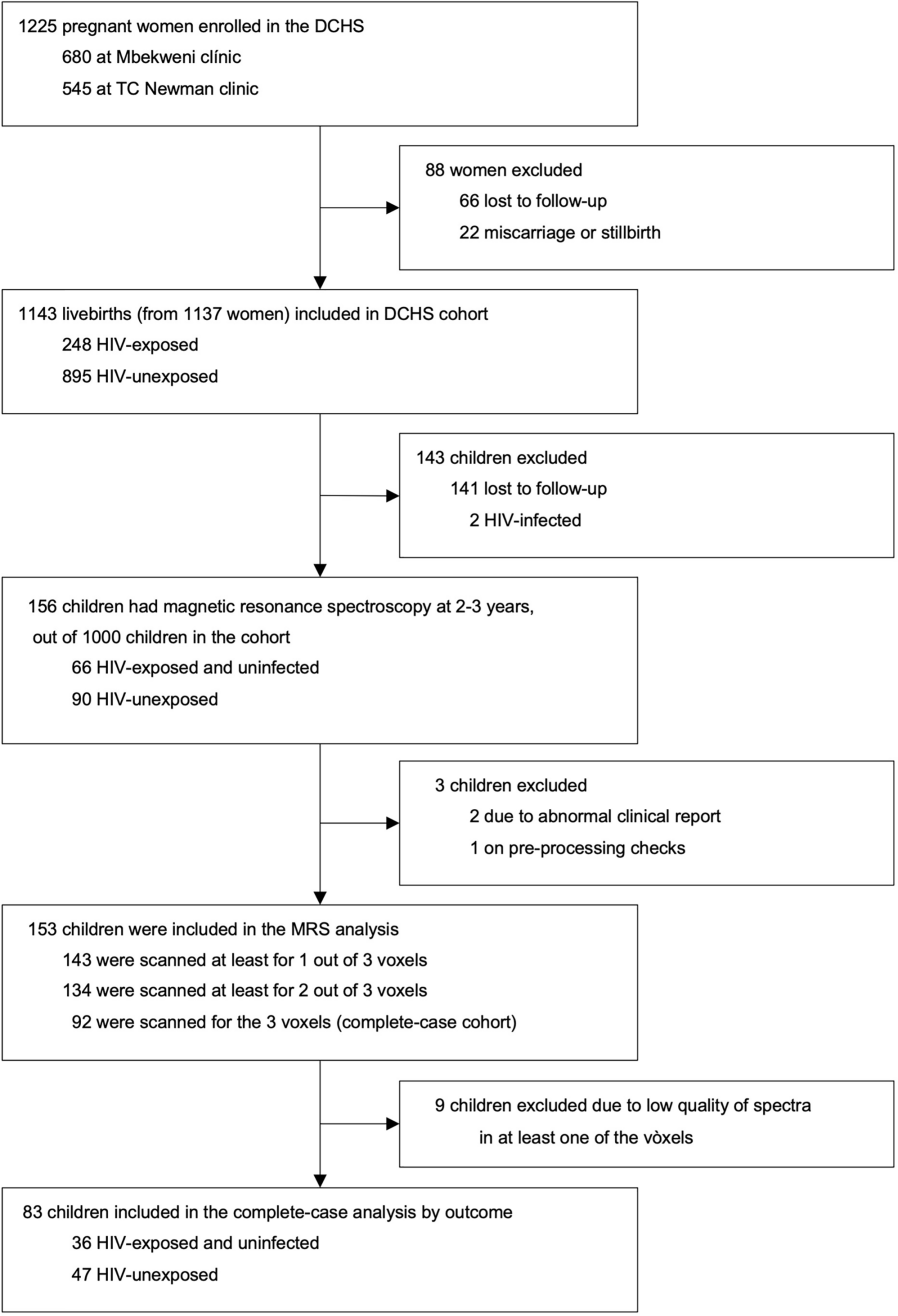
DCHS flowchart for MRS sub-study. Exclusion criteria for the MRS nested sub-study are described in the methodology section.

A total of 1143 mother-child pairs were enrolled in the DCHS. A subset of 156 children had MRS imaging at age 2-3 years. Of these, 143 had a successful MRS acquisition from the parietal grey matter voxel (first voxel in the data acquisition protocol), 134 from the left parietal WM voxel (acquired second), and 92 from the right parietal WM voxel (acquired third and last). A total of 9 participants were excluded from the study after inspection of obtained MRS data due to low quality of spectra in at least one of the three voxels. Our final complete-case cohort included 83 children (36 CHEU, 47 CHU) who had usable metabolite data for all three voxels (i.e., GM, left and right WM) and complete covariate data (**Figure 2**).

Socioeconomic characteristics of the complete-case cohort of children were comparable between groups. Mothers living with and without HIV had similar household incomes, education, employment status, marital status, hospitalization rates and smoking or alcohol use during pregnancy (**Table 1**). However, mothers living with HIV were older at delivery and, among those with available data (N=28 CHEU, N=42 CHU), there were lower rates of depression compared to their uninfected counterparts. Weight at birth was similar for CHEU and CHU. Exclusive breastfeeding duration was comparable between groups, as was the proportion of children with WHO markers for poor nutrition. All mothers living with HIV received first-line three-drug ART regimens, whereas post-exposure prophylaxis for CHEU included nevirapine (77.7%) or nevirapine and zidovudine (22.3%). The complete-case cohort and the original subset of 156 children were similar in terms of sociodemographic characteristics (**Supplementary Table 1**).

**Table 1:** Sociodemographic characteristics of children included in the MRS complete-case analysis, according to HIV exposure.

	CHEU (N = 36)	CHU (N = 47)	p value
	Mean (±SD) or n/N (%)	Mean (SD) or n/N (%)	
**Child age at scan (in months)**	33.78 (±1.83)	34.15 (±1.75)	0.35
**Sex**			0.14
Male	25/36 (69.44%)	24/47 (51.06%)	
Female	11/36 (30.55%)	23/47 (48.93%)	
**Monthly household income (in ZAR)**			0.49
< 1000	12/36 (33.33%)	17/47 (37.17%)	
1000 - 5000	23/36 (63.88%)	26/47 (55.31%)	
> 5000	1/36 (2.77%)	4/47 (8.51%)	
**Maternal education**			0.82
Primary	3/36 (8.33%)	3/47 (6.38%)	
Some secondary	22/36 (61.11%)	26/47 (55.31%)	
Completed secondary	10/36 (27.77%)	15/47 (31.91%)	
Tertiary	1/36 (2.77%)	3/47 (6.38%)	
**Employed mother**	9/36 (25%)	9/47 (19.14%)	0.70
**Maternal relationship status (partnered)**	19/35 (54.28%)	17/47 (36.17%)	0.22
**Maternal age at delivery (in years)**	29.89 (±4.37)	25.65 (±5.06)	0.0001*
**Gestational age at delivery (in weeks)**	38.61 (±2.27)	38.85 (±2.86)	0.67
**Premature birth (< 37 weeks’ gestation)**	5/36 (13.88%)	6/47 (12.76%)	1.00
**Birthweight (in g)**	3030 (±501.76)	3132 (±622.48)	0.40
**Nutritional status at 2 years old**			
Stunting (height-for-age Z-score < -2)	5/31 (16.13%)	5/41 (12.19%)	0.89
Underweight (weight-for-age Z-score < -2)	2/31 (6.45%)	1/41 (2.44%)	0.80
Wasting (weight-for-length Z-score < -2)	0/31 (0%)	0/41 (0%)	–
**Maternal hospitalization during pregnancy**	3/36 (8.33%)	4/47 (8.51%)	1.00
**Maternal smoking during pregnancy**	7/36 (19.44)	11/46 (23.91)	0.67
**Maternal alcohol use during pregnancy**	3/35 (8.57%)	10/46 (21.74%)	0.20
**Maternal depression during pregnancy**	1/28 (3.57%)	11/42 (26.19%)	0.032*
**Exclusive breastfeeding duration (in months)**	1.919 (±2.25)	2.180 (±1.47)	0.54
**Maternal HIV diagnosis timepoint**			
Before pregnancy	26/36 (72.22%)		
During pregnancy	10/36 (27.77%)		
**Maternal lowest CD4 cell count^§^** **during pregnancy**			
≤ 500 cells/mm^3^	12/26 (46.15%)		
> 500 cells/mm^3^	14/26 (53.85%)		
**Highest maternal viral load during pregnancy**			
(undetectable) < 40 copies/ml	25/29 (86.20%)		
40 - 1000 copies/ml	2/29 (6.90%)		
>1000 copies/ml	2/29 (6.90%)		
**Antiretroviral therapy initiation**			
Before pregnancy	20/36 (55.55%)		
During pregnancy	16/36 (44.44%)		
**First-line antiretroviral therapy during pregnancy**			
Fixed dose combination (Efavirenz+ Emtricitabine + Tenofovir)	33/36 (91.66%)		
Lamivudine + Zidovudine + Nevirapine	2/36 (5.55%)		
Lamivudine + Zidovudine + Efavirenz	1/36 (2.77%)		
**Infant prophylaxis**			
Nevirapine alone	28/36 (77.77%)		
Nevirapine and zidovudine	8/36 (22.22%)		

Data are mean (±SD) or n/N (%). *p<0.05. Percentages calculated out of available data. Continuous data was assessed for normality using Shapiro-Wilk tests. Comparisons between CHEU and CHU were made using t-tests or Wilcoxon tests for normally and non-normally distributed continuous data, respectively, and X^2^ tests with Yates correction for categorical data. Missing data: maternal relationship status (N = 1 in the CHEU group); nutritional conditions at 2 years old (N = 5 in the CHEU group, N = 6 in the CHU group); maternal smoking during pregnancy (N = 1 in the CHU group); maternal alcohol use during pregnancy (N = 1 in the CHEU group, N = 1 in the CHU group); maternal depression during pregnancy (N = 8 in the CHEU group, 5 in the CHU group); maternal CD4 cell count in pregnancy (N = 10); highest maternal viral load during pregnancy (N = 7). **^§^** The lowest maternal CD4 cell count within 1 year before birth and 3 months after birth was used to maximise numbers. CHEU, children who are HIV-exposed and uninfected; CHU, children who are HIV-unexposed; ZAR, South African Rand; WHO, World Health Organization.

### Metabolite Patterns of CHEU and CHU

Fractional tissue composition in each of the three voxels of the complete-case cohort did not differ between groups. The percentage of GM in the voxel targeted at parietal GM was ≈77% for both CHEU and CHU, while the voxels targeted at left and right parietal WM contained ≈52% of WM in both groups (**Table 2**). For all spectral fits the CRLB for NAA/Cr+PCr were ≤7%, for Ins/Cr+PCr ≤6%, for GPC+PCh/Cr+PCr ≤6%, and for Glu/Cr+PCr ≤8%.

**Table 2:** Fractional tissue composition in each defined MRS voxel, according to HIV exposure.

Voxel	CHEU (N = 36)			CHU (N = 47)		
	% Grey matter	% White Matter	% CSF	% Grey matter	% White Matter	% CSF
Parietal grey matter	**77.9** (±4.2)	12.9 (±2.8)	9.2 (±3.2)	**77.2** (±4.5)	14.1 (±2.8)	8.7 (±2.9)
Left parietal white matter	45.2 (±8.8)	**52.1** (±8.9)	2.7 (±1.6)	46.8 (±7.0)	**51.1** (±7.5)	2.1 (±1.2)
Right parietal white matter	46.2 (±8.7)	**51.9** (±9.2)	1.9 (±1.3)	46.1 (±6.6)	**52.5** (±7.0)	1.4 (±0.8)

Data is displayed as mean (±SD) percentages. Bold percentages indicate targeted tissue in each voxel. Data was assessed for normality using Shapiro-Wilk tests. Comparisons between CHEU and CHU were made using t-tests or Wilcoxon tests for normally and non-normally distributed data, respectively. All p values were greater than 0.05 (data not shown). CHEU, children who are HIV-exposed and uninfected; CHU, children who are HIV-unexposed; CSF, cerebrospinal fluid.

Bartlett sphericity and KMO tests confirmed the factorability of our data. Subsequent factor analysis identified four factors (RMSEA < 0.05), which accounted for 69% of data variability (**Table 3**). Each factor is a metabolic pattern composed of loadings associated with each of the metabolite ratios (/Cr+PCr), where a large loading (>0.6) indicates a strong contribution of a certain metabolite ratio to the factor. Factor 1 was composed of large loadings of NAA/Cr+PCr across all three brain regions and a strong contribution of Glu/Cr+PCr in the voxel targeted at parietal GM. Factor 2 was dominated by large loadings of Ins/Cr+PCr across brain regions. Factor 3 was composed of large loadings of GPC+PCh/Cr+PCr in the voxels targeted at left and right parietal WM, and a medium contribution (0.552) of the same metabolite in the voxel targeted at parietal GM. Factor 4 was characterized by large loadings of Glu/Cr+PCr in the voxel targeted at right parietal WM and a medium contribution (0.530) of the same metabolite ratio in the voxel targeted at left parietal WM.

**Table 3:** Factor loadings.

Voxel	Metabolite	Factor Loading			
		Factor 1	Factor 2	Factor 3	Factor 4
**PGM**	**Glu/Cr+PCr**	**0.745**	-0.044	0.036	0.314
	**Ins/Cr+PCr**	-0.111	**0.767**	0.062	-0.208
	**NAA/Cr+PCr**	**0.911**	-0.145	-0.052	0.025
	**GPC+PCh/Cr+PCr**	-0.264	0.211	**0.552**	0.007
**LPWM**	**Glu/Cr+PCr**	0.439	-0.034	0.100	**0.530**
	**Ins/Cr+PCr**	-0.182	**0.906**	0.015	0.003
	**NAA/Cr+PCr**	**0.889**	-0.116	0.053	0.159
	**GPC+PCh/Cr+PCr**	0.113	-0.086	**0.821**	0.131
**RPWM**	**Glu/Cr+PCr**	0.151	0.008	-0.043	**0.883**
	**Ins/Cr+PCr**	-0.111	**0.823**	0.001	0.168
	**NAA/Cr+PCr**	**0.692**	-0.208	-0.029	0.072
	**GPC+PCh/Cr+PCr**	0.104	-0.005	**0.862**	-0.115

Bartlett sphericity and Kaiser-Meyer-Olkin tests were performed and confirmed that a factor analysis approach was suitable for our data. Factor analysis identified four main metabolic patterns (RMSEA < 0.05), which accounted for 69% of data variability and are displayed in this table. Factor loadings in bold represent the main components of each metabolic pattern.

PGM, parietal grey matter; LPWM, left parietal grey matter; RPWM, right parietal white matter; NAA, n-acetyl-aspartate; Ins, myo-inositol; GPC+PCh, total choline (glycerophosphocholine + phosphocholine); Glu, glutamate;/Cr+PCr, relative to creatine + phosphocreatine.

In both unadjusted and adjusted logistic regression models, HIV exposure was significantly predicted by factor 2 (dominated by Ins/Cr+PCr across regions), with an OR estimate of 1.63 (95% CI 1.11 - 2.50) and adjusted OR 1.59 (95% CI 1.07 - 2.47), respectively (**Table 4**). None of the remaining three factors predicted HIV exposure. Sensitivity analyses revealed similar results when separately adjusting for maternal age at delivery, maternal depression during pregnancy and infant birthweight, with HIV exposure being significantly predicted by factor 2 (**Supplementary Table 2**).

**Table 4:** Logistic regression analysis of factor scores as predictors for HIV exposure.

	Mean factor score		Unadjusted logistic regression			Adjusted logistic regression*		
	CHEU(N = 36)	CHU(N = 47)	OR	Confidence interval (95%)	P value	OR	Confidence interval (95%)	P value
**Factor 1** (**NAA**)	-0.182	0.139	0.72	0.45 – 1.12	0.14	0.72	0.44 – 1.50	0.18
**Factor 2** (**Ins**)	**0.368**	**-0.282**	**1.63**	**1.11** – **2.50**	**0.017**	**1.59**	**1.07** – **2.47**	**0.029**
**Factor 3** (**GPC+PCh**)	-0.030	0.023	0.91	0.51 – 1.59	0.80	0.82	0.42 – 1.55	0.54
**Factor 4** (**Glu**)	0.097	-0.074	1.28	0.76 – 2.21	0.35	1.41	0.81 – 2.56	0.23

Odds ratios (OR) greater than 1 indicate an increased likelihood of association between a certain metabolite pattern and HIV exposure. Bold data represents statistically significant associations. *Adjusted for child age, child sex, and maternal alcohol use during pregnancy.

NAA, metabolite pattern dominated by n-acetyl-aspartate ratios; Ins, metabolite pattern dominated by myo-inositol ratios; GPC+PCh, metabolite pattern dominated by total choline (glycerophosphocholine + phosphocholine) ratios; Glu, metabolite pattern dominated by glutamate ratios; CHEU, children who are HIV-exposed and uninfected; CHU, children who are HIV-unexposed.

### Region-Specific Relative Concentrations of Metabolites to Total Creatine in CHEU and CHU

**Figure 3:**
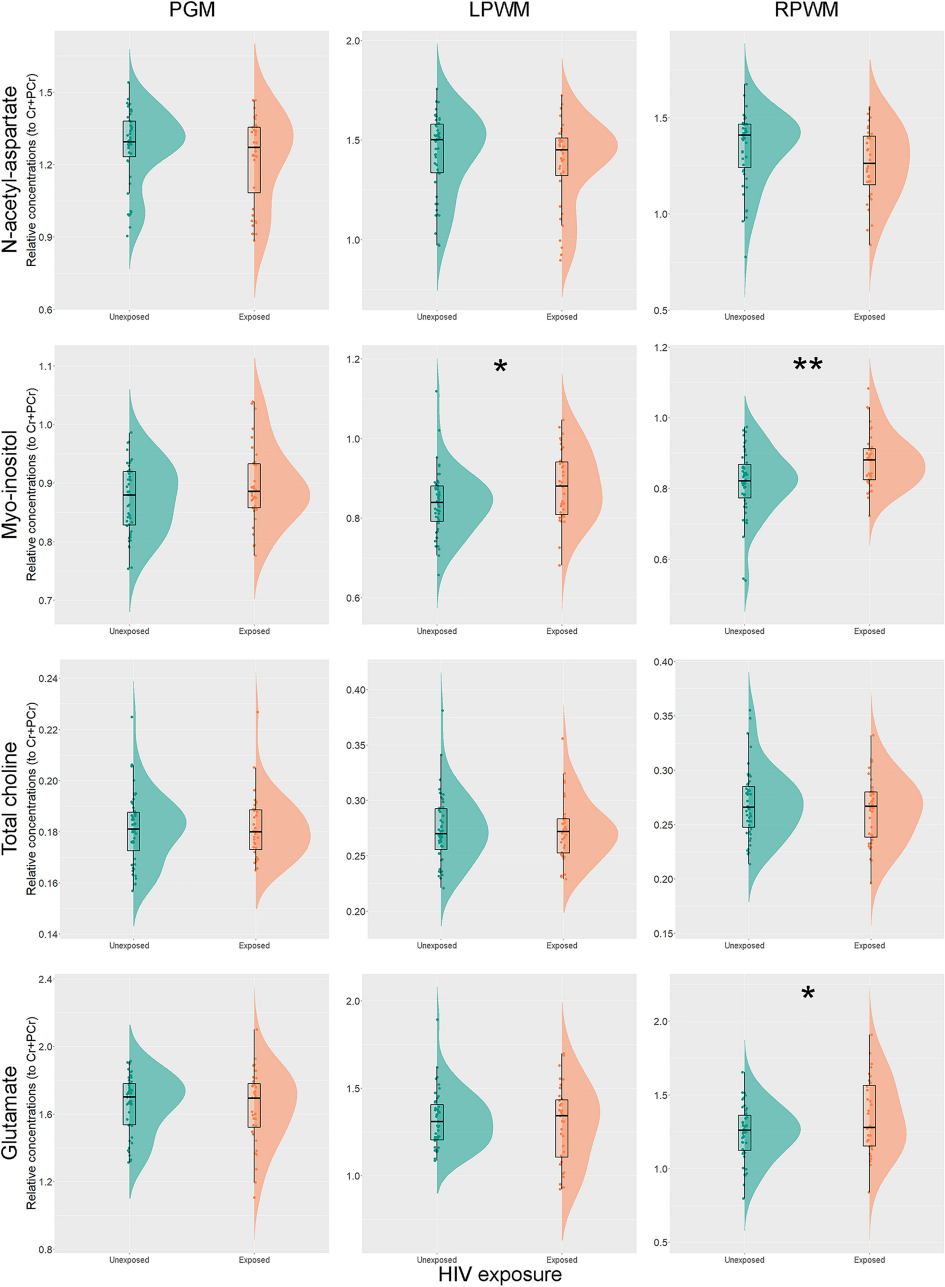
Individual metabolite relative concentrations. Raincloud plots (54) showing individual metabolite relative concentrations to total creatine in the parietal grey matter (PGM), left parietal white matter (LPWM) and right parietal white matter (RPWM) in our complete-case cohort, according to HIV exposure. *p<0.05; **p<0.01.

Unadjusted analyses for each individual metabolite relative concentration to total creatine and brain region revealed significantly higher ratios of Ins/Cr+PCr in left (p = 0.025) and right parietal WM (p = 0.001) of CHEU, compared to their unexposed peers. Levels of Glu/Cr+PCr in the right parietal WM of CHEU were also significantly higher than those of CHU (p = 0.034) (**Figure 3** and **Supplementary Table 3**).

The adjusted analyses did not substantially modify the results obtained for Ins/Cr+PCr (p = 0.004) and Glu/Cr+PCr (p = 0.015) in the right parietal WM, while group differences in Ins/Cr+PCr (p = 0.066) in the left parietal WM fell short of our selected threshold for statistical significance. Results remained similar for all metabolite ratios after accounting for the percentage of GM in WM voxels (data not shown).

### Association of Maternal Immune Status and ART Initiation With Child Metabolite Patterns

Maternal immune status and ART initiation variables were found to be co-linear in this sub-group (correlation coefficient >0.7, Cramér’s V test). Further, given only 72% mothers of CHEU children in this sample had CD4 cell counts taken during pregnancy, we were unable to run multinomial logistic regression using these variables as due to small sample size and missing data we recognized that our ability to draw valid conclusions from this analysis would be limited.

## Discussion

Our study is the first to describe the impact of HIV exposure without infection on brain metabolites at 2-3 years of age in a well-characterised cohort of children living in a LMIC setting. By combining MRS data from parietal grey and white matter regions using a factor analysis approach, we demonstrate a neurometabolite pattern of elevated Ins/Cr+PCr in the parietal brain regions of CHEU; this elevation is suggestive of neuroinflammatory processes.

Factor analysis identified four metabolic patterns in the parietal brain regions of our young cohort. Although all factors represent a weighted combination of all metabolite ratios to total creatine in each region, each factor was characterized by large contributions from a certain metabolite ratio grouped across brain regions with generally small contributions from the other metabolite ratios. Based on prior studies of paediatric MRS (32, 33), we proposed the following interpretations: Factor 1 was interpreted as a metabolic pattern for neuronal health or integrity, due to high loadings of NAA/Cr+PCr across brain regions. It also contained a strong contribution from Glu/Cr+PCr in parietal grey matter, suggesting that glutamate may covary with n-acetyl-aspartate in certain regions and therefore with number or density of neurons. Factor 2 (dominated by Ins/Cr+PCr loadings across all regions) was considered an inflammatory pattern for neuroinflammation or gliosis; and Factor 3 (characterized by GPC+PCh/Cr+PCr across brain regions) was interpreted as a pattern for membrane maturation (32, 33). Factor 4 was dominated by Glu/Cr+PCr across WM regions. This made it challenging to assign an interpretation distinct from that of Factor 1. However, given the role of glutamate in neurocognitive processes including memory, sensory and motor processing (see Blüml et al. and references) (33), Factor 4 was broadly interpreted as a pattern for neuronal function.

We found the inflammatory pattern was associated with HIV exposure, both in the unadjusted and adjusted logistic regression models. In the neurotypical brain, levels of the glial marker Ins/Cr+PCr reach final, stable values within the first year of life (32). Therefore, a pattern of covarying Ins/Cr+PCr across brain regions at 2-3 years of age suggests neurometabolic development in CHEU may be influenced by underlying neuroinflammatory processes. Of note, maternal alcohol use during pregnancy did not substantially modify the results of the unadjusted analysis, despite its described association with lower glutamate concentrations in the parietal WM in neonates (50).

While there are no previous MRS reports of CHEU at this age, neurometabolic differences in this population have been reported in older children. Low absolute concentrations of creatine and phosphocreatine, n-acetyl-aspartate, total choline, and glutamate were found in the basal ganglia of a South African cohort of CHEU at age 9 years, compared to their unexposed peers, indicating possible neuronal damage (35). A longitudinal analysis of the same cohort found no interactions between age and HIV exposure when exploring neurometabolic development from 5 to 10 years of age (34). Further, at age 11 years, lower absolute concentrations of n-acetyl-aspartate were observed in frontal GM and peritrigonal WM of CHEU, suggesting possible axonal damage (36). Taken together, these results reflect the dynamic nature of neurometabolic development across child ages and brain regions, and the importance of analysing neurometabolites at different ages. However, children at older ages may have been exposed to additional sociodemographic and psychosocial risk factors that may impact their brain development adding a layer of complexity to the interpretation of results. Our study has the advantage of exploring neurometabolic development at a younger age, minimising the influence of socioenvironmental confounders.

Ins/Cr+PCr was significantly higher in left and right parietal WM of CHEU in our unadjusted analysis, and right parietal WM differences remained significant after adjusting for child age, child sex, and maternal alcohol use during pregnancy. WM may therefore be particularly sensitive to neuroinflammation from HIV exposure. Altered WM microstructural development has previously been reported in the right posterior corona radiata and the corticospinal tract of CHEU at age 7 years compared to CHU (31), and in neonates from the DCHS in the middle cerebellar peduncles (29) supporting our findings.

In addition to our main finding of higher parietal Ins/Cr+PCr in CHEU, we found differences in other metabolite ratios between groups. Glu/Cr+PCr levels were higher in the right parietal WM of CHEU in both unadjusted and adjusted analyses, compared to CHU. While covarying levels of Glu/Cr+PCr in WM were considered a pattern for neuronal function in our factor analysis, in the context of HIV exposure and neuroinflammation glial cells are primed and may fail to regulate glutamate. This has been demonstrated in patients with brain injuries or neuropsychiatric disorders, resulting in an unusual increase of this neurotransmitter in the extracellular space (55–57), which may also explain our results here. No results were modified after adjusting for GM percentage in voxels targeted at parietal WM in our sensitivity analyses, despite the presence of this confounder in the composition of such voxels.

Overall, our findings of increased Ins/Cr+PCr in the WM of CHEU add to the literature that HIV exposure may impact on WM development by affecting underlying neuroinflammatory processes. Animal model studies suggest that maternal immune activation induces exaggerated neuroinflammatory processes in offspring (19, 20). One of the main reported effects is microglial priming, where microglial cells become prone to produce an exaggerated response against second hits (18). Therefore, postnatal threats such as infections or environmental stressors, may elicit a neuroinflammatory overreaction in the young brain with long-term consequences (18–20). *In utero* priming of the immune system may take place in CHEU (21–23), and of note, inflammatory metabolite patterns of myo-inositol and total choline have been associated with cognitive impairment in adults (37, 38, 58) and children (35, 59) living with HIV.

Psychosocial variables may also play a key role in the neurometabolic development of CHEU. In LMICs studies, maternal depression and alcohol use during pregnancy have separately been associated with poorer cognitive outcomes in this population (8, 60). A recent US study linked maternal depression with decreased creatine and phosphocreatine, n-acetyl-aspartate, and total choline levels in the developing brain of HIV-unexposed foetuses (61) suggesting maternal immune activation may play a role (62). We found the impact of HIV exposure on Ins/Cr+PCr was independent of maternal depression and alcohol use in pregnancy. However, whether the neurobiological mechanisms underpinning these factors overlap with those derived from HIV exposure needs to be determined in larger samples. Separately, infant birthweight has been associated with maternal HIV infection (63). Although, studies are heterogeneous, suggesting the relationship between maternal HIV status and infant birthweight may vary across settings (51). Given birthweight may be influenced by maternal immune activation during pregnancy (64) and has been reported to impact children’s performance in developmental assessments at 2 years of age (8), we examined infant birthweight in sensitivity analyses and found this did not modify our results.

HIV-specific factors have also been found to impact CHEU outcomes, including maternal CD4 and ART. In a sub-study of CHEU from the South African CHER cohort, lower CD4/CD8 ratio in infancy correlated to lower basal ganglia n-acetyl-aspartate and total choline levels at 5 years (65), lower total choline levels at 7 years, and lower myo-inositol levels at 9 years of age (35). The results suggest that an altered immune status in infancy may be associated with poorer neuronal and glial cell density in childhood. Since long-term ART exposure has been linked to mitochondrial toxicity in the brain (24, 66), MRS could also be used in CHEU to measure mitochondrial markers, such as lactate (32, 33). Although we were limited in our ability to examine maternal CD4 and ART in this sample, future studies may examine the relationship between these variables and neurometabolites in CHEU.

The strengths of our study include the use of a robust approach to study the effect of HIV exposure on neurometabolic development at 2-3 years of age, comparing a well-characterized sample of CHEU to an appropriate control group with similar sociodemographic characteristics from a LMIC setting. Overall, our findings provide novel information about the neurobiological profile of young CHEU in a sub-Saharan African setting. We performed robust sensitivity analyses which did not substantially modify the results obtained in the adjusted logistic regression model. Furthermore, our cohort had a high prevalence of sociodemographic and psychosocial risk factors, comparable to other LMICs, and, all mothers living with HIV in our cohort received first-line triple ART, the majority with a fixed dose combination of efavirenz, emtricitabine, and tenofovir, implying our cohort may have generalisability for other CHEU populations across sub-Saharan Africa.

This study has some limitations to consider in the interpretation of our findings. First, MRS in very young paediatric subjects is technically challenging, since lack of motion is essential for successful data acquisition. As some data were lost due to children motion, the size of our complete-case cohort for analysis was substantially reduced, resulting in potential for underpowering of our analysis. However, sociodemographic characteristics were similar between the complete-case cohort and the full neuroimaging cohort, minimizing the likelihood of selection bias. This reduction in sample size meant we were unable to explore the association of maternal CD4 cell counts during pregnancy with child metabolite patterns, which needs to be investigated in future work. Second, our study design only included voxels placed in the parietal regions of the developing brain, so we are unable to draw conclusions about the presence of an inflammatory pattern in other brain areas of CHEU. Third, since WM is still under maturation in the developing brain (67), the tissue composition of voxels targeted at parietal WM may have included a proportion of GM. Hence, we cannot claim metabolite ratios obtained from these voxels purely belong to WM tissue. To mitigate this limitation, we ran sensitivity analyses for region-specific comparisons of individual metabolite ratios between groups, adjusting for GM percentage in voxels targeted at parietal WM, and found our results held. Fourth, although total creatine is well characterized and stable in the neurotypical brain during the first years of life (32, 33), low levels of this reference have been described in the peritrigonal WM in children living with HIV (36), compared to CHEU and CHU, and in subcortical brain regions in CHEU, compared to CHU (35). In contrast, higher creatine levels have been described in the parietal WM in adult subjects living with HIV, compared to uninfected peers (37). Therefore, although relative concentrations are commonly reported as they have the advantage of being less dependent on correction for relaxation and partial volume effects compared to absolute concentrations, the use of creatine and phosphocreatine as a reference in CHEU studies complicated interpretation as findings may reflect a change in the numerator or denominator. Similarly, the roles of metabolites in the developing brain, particularly n-acetyl-aspartate, remain to be fully established and Factor interpretations should be viewed with some caution. Lastly, we did not correct for multiple comparisons in our analyses, given the exploratory nature of our study and our use of factor analysis as a dimensionality-reduction method to reduce comparisons. Further work will be needed in larger sample sizes to replicate results.

In conclusion, our study presents the first results of the neurometabolic impact of HIV exposure in children from a LMIC setting during their first 2-3 years of life. We report differences in brain metabolite patterns between CHEU and CHU, showing an association of HIV exposure with an inflammatory pattern of elevated Ins/Cr+PCr in parietal brain regions. Our results are suggestive of neuroinflammatory processes in the developing brain of CHEU at this early age, which may be especially relevant in the parietal WM; whether this represents a potential target for specific neurodevelopmental interventions remains to be determined. Future work is needed to assess the longitudinal trajectories of neurometabolites in the population of CHEU, and to investigate associations with neurocognitive development and mechanisms underlying the inflammatory profile.

## Data Availability Statement

The original contributions presented in the study are included in the article/**Supplementary Material**. Further inquiries can be directed to the corresponding author.

## Ethics Statement

The studies involving human participants were reviewed and approved by the Faculty of Health Sciences, Human Research Ethics Committee, University of Cape Town (401/2009; 525/2012 & 044/2017), by Stellenbosch University (N12/02/0002), and by the Western Cape Provincial Health Research committee (2011RP45). Written informed consent to participate in this study was provided by the participants’ legal guardian/next of kin.

## Author Contributions

CB-C: methodology, formal analysis and interpretation, visualization, writing – original draft, and review & editing. CW: conceptualization, investigation, data curation, supervision, and writing - review & editing. FR: methodology, formal analysis, supervision, and writing – review & editing. SS: investigation and writing – review & editing. KN: methodology and writing – review & editing. SJ: methodology and writing – review & editing. ARo: investigation and writing – review & editing. NH: project administration and writing – review & editing. ARe: formal analysis and writing – review & editing. HZ: conceptualization, methodology, resources, and writing - review & editing. DS: conceptualization, methodology, resources, and writing – review & editing. KD: conceptualization, methodology, investigation, resources, supervision, and writing – review & editing. All authors approved the final version.

## Funding

The Drakenstein Child Health Study is funded by the Bill and Melinda Gates Foundation (OPP 1017641), the NRF and the NIH. Additional support for HZ and DS was provided by the Medical Research Council of South Africa. CW was supported by the Wellcome Trust (203525/Z/16/Z). KD and aspects of the research are additionally supported by the NRF, an Academy of Medical Sciences Newton Advanced Fellowship (NAF002/1001) funded by the UK Government’s Newton Fund, by NIAAA *via* (R21AA023887), by the Collaborative Initiative on Fetal Alcohol Spectrum Disorders (CIFASD) developmental grant (U24 AA014811), and by the US Brain and Behaviour Foundation Independent Investigator grant (24467). ARe is partially funded by the UK Medical Research Council (MRC) and the UK Foreign, Commonwealth and Development Office (FCDO) under the MRC/DFID Concordat agreement which is also part of the EDCTP2 programme supported by the European Union, Grant Ref: MR/R010161/1.

## Conflict of Interest

The authors declare that the research was conducted in the absence of any commercial or financial relationships that could be construed as a potential conflict of interest.

## Publisher’s Note

All claims expressed in this article are solely those of the authors and do not necessarily represent those of their affiliated organizations, or those of the publisher, the editors and the reviewers. Any product that may be evaluated in this article, or claim that may be made by its manufacturer, is not guaranteed or endorsed by the publisher.
