# Supplementary material for: A Neurometabolic Pattern of Elevated Myo-Inositol in Children Who Are HIV-Exposed and Uninfected: A South African Birth Cohort Study
Source: Front Immunol. 2022 Mar 28;13:800273. doi: 10.3389/fimmu.2022.800273 (PMC8995436; doi:10.3389/fimmu.2022.800273)
Supplement: Supplementary file 1 [file Table_1.pdf]

Supplementary Material

Supplementary Table 1 | Sociodemographic characteristics of the complete-case cohort of children included in the MRS analysis, compared to all children with MRS scans.

	**Complete-case cohort (N = 83)**	**Original cohort (N = 156)**	
	Mean (±SD) or n/N (%)	Mean (SD) or n/N (%)	**p value**
**Child age at scan (in months)**	33.99 (± 1.79)	33.96 (± 1.77)	0.71
**Sex**			0.91
Male	49/83 (59.04%)	91/156 (58.33%)	
Female	34/83 (40.96%)	65/156 (41.67%)	
**Monthly household income (in ZAR)**			0.70
< 1000	29/83 (34.94%)	48/156 (30.77 %)	
1000 - 5000	49/83 (59.04%)	95/156 (60.90 %)	
> 5000	5/83 (6.02%)	13/156 (8.33 %)	
**Maternal education**			0.96
Primary	6/83 (7.23%)	9/156 (5.77%)	
Some secondary	48/83 (57.83%)	92/156 (58.97%)	
Completed secondary	25/83 (30.12%)	46/156 (29.49%)	
Tertiary	4/83 (4.82%)	9/156 (5.77%)	
**Maternal employment**	18/83 (21.68%)	42/156 (26.92%)	0.37
**Maternal relationship status (partnered)**	36/82 (43.90%)	74/155 (47.74%)	0.57
**Maternal age at delivery (in years)**	27.49 (± 5.19)	27.94 (± 5.75)	0.68
**Gestational age at delivery (in weeks)**	38.75 (± 2.61)	38.79 (± 2.40)	0.99
**Premature birth (< 37 weeks' gestation)**	11/83 (13.25%)	21/156 (13.46%)	0.96
**Birthweight (in g)**	3088 (± 572.24)	3144 (± 562.67)	0.87
**Nutritional conditions at 2 years old (based on WHO Z scores guidelines)**			

Stunting (height-for-age Z-score < -2)	10/72 (13.88%)	28/137 (20.44%)	0.24
Underweight (weight-for-age Z-score < -2)	3/72 (4.16%)	8/137 (5.84%)	0.60
Wasting (weight-for-length Z-score < -2)	0/72 (0.00%)	1/137 (0.73%)	-
**Maternal hospitalization during pregnancy**	7/83 (8.43%)	10/154 (6.49%)	0.58
**Maternal smoking during pregnancy**	18/81 (22.22%)	29/151 (17.22)	0.59
**Maternal alcohol use during pregnancy**	13/81 (16.05%)	23/127 (18.11%)	0.70
**Maternal depression during pregnancy**	12/70 (17.14%)	31/129 (24.03%)	0.26
**Exclusive breastfeeding duration (in months)**	2.06 (± 1.84)	1.83 (± 1.79)	0.30
**Maternal HIV status**			0.87
Positive	36/83 (43.37%)	66/156 (42.31%)	
Negative	47/83 (56.63%)	90/156 (57.69%)	

Data are mean (± SD) or n/N (%). Percentages calculated out of available data. Continuous data was assessed for normality using Shapiro-Wilk tests. Comparisons between CHEU and CHU were made using *t*-tests or Wilcoxon tests for normally and non-normally distributed continuous data, respectively, and X^2 tests with Yates correction for categorical data.

Missing data: maternal relationship status (N = 1 in the complete-case cohort, N = 1 in the original cohort); nutritional conditions at 2 years old (N = 11 in the complete-case cohort, N = 19 in the original cohort); maternal hospitalization during pregnancy (N = 2 in the original cohort); maternal smoking during pregnancy (N = 2 in the complete-case cohort, N = 5 in the original cohort); maternal alcohol use during pregnancy (N = 2 in the complete-case cohort, N = 29 in the original cohort); maternal depression during pregnancy (N = 13 in the complete-case cohort, N = 27 in the original cohort).

Supplementary Table 2 | Sensitivity analyses for maternal age of delivery, maternal depression during pregnancy, and infant birthweight.

	Sensitivity analysis for maternal age at delivery*			Sensitivity analysis for maternal depression**			Sensitivity analysis for infant birthweight***		
	**OR**	**Confidence interval** (95%)	**P value**	**OR**	**Confidence interval** (95%)	**P value**	**OR**	**Confidence interval** (95%)	**P value**
**Factor 1** (**NAA**)	0.62	0.34 – 1.00	0.09	0.63	0.35 – 1.08	0.10	0.76	0.03 – 2.97	0.24
**Factor 2** (**Ins**)	**1.82**	**1.17 – 3.02**	**0.012**	**1.65**	**1.04 – 2.82**	**0.045**	**1.56**	**1.05 – 2.44**	**0.036**
**Factor 3** (**GPC+PCh**)	0.94	0.45 – 1.88	0.86	1.10	0.54 – 2.31	0.79	0.79	0.39 – 1.50	0.47
**Factor 4** (**Glu**)	1.26	0.69 – 2.34	0.45	1.14	0.59 – 2.27	0.69	1.46	0.82 – 2.68	0.20

Logistic regression analysis of factor scores as predictors for HIV exposure. Odds ratios (OR) greater than 1 indicate an increased likelihood of association between a specific metabolite pattern and HIV exposure. Bold data represents values belonging to statistically significant associations ($p<0.05$). *Adjusted for child age, child sex, maternal alcohol use during pregnancy and maternal age at delivery. **Adjusted for child age, child sex, maternal alcohol use during pregnancy and maternal depression during pregnancy. ***Adjusted for child age, child sex, maternal alcohol use during pregnancy and infant birthweight.

NAA: metabolite pattern dominated by n-acetyl-aspartate ratios; Ins: metabolite pattern dominated by myo-inositol ratios; GPC+PCh: metabolite pattern dominated by total choline (glycerophosphocholine + phosphocholine) ratios; Glu: metabolite pattern dominated by glutamate ratios.

Supplementary Table 3 | Comparison of region-specific metabolite ratios between CHEU and CHU groups.

	Parietal grey matter						Left parietal white matter						Right parietal white matter					
	Unadjusted			Adjusted*			Unadjusted			Adjusted*			Unadjusted			Adjusted*		
**Metabolite ratios**	**β**	**Robust SE**	**p-value**	**β**	**Robust SE**	**p-value**	**β**	**Robust SE**	**p-value**	**β**	**Robust SE**	**p-value**	**β**	**Robust SE**	**p-value**	**β**	**Robust SE**	**p-value**
**NAA/Cr+PCr**	-0.140	0.037	0.21	-0.174	0.040	0.15	-0.117	0.046	0.29	-0.109	0.050	0.36	-0.212	0.041	0.054	-0.231	0.036	0.07
**Ins/Cr+PCr**	0.091	0.016	0.42	0.112	0.019	0.34	**0.246**	**0.018**	**0.025**	0.223	0.020	0.066	**0.335**	**0.018**	**0.001**	**0.329**	**0.021**	**0.004**
**GPC+PCh/Cr+PCr**	0.076	0.003	0.50	0.065	0.003	0.58	-0.013	0.006	0.90	-0.060	0.005	0.58	-0.107	0.006	0.328	-0.143	0.006	0.22
**Glu/Cr+PCr**	-0.053	0.044	0.63	-0.026	0.047	0.83	-0.073	0.043	0.52	-0.043	0.045	0.71	**0.241**	**0.048**	**0.034**	**0.287**	**0.051**	**0.015**

In unadjusted and adjusted analyses, comparisons between CHEU (N = 36) and CHU (N = 47; reference group) were made using linear regression analyses with robust standard errors. *Adjusted for child age, child sex, and maternal alcohol use during pregnancy. Bold data represents values belonging to statistically significant associations ($p<0.05$).

β: standardized coefficient; SE: standard error; NAA: n-acetyl-aspartate; Ins: myo-inositol; GPC+PCh: total choline (glycerophosphocholine + phosphocholine); Glu: glutamate; /Cr+PCr: relative to creatine + phosphocreatine.
